# Antibacterial Hydrophilic ZnO Microstructure Film with Underwater Oleophobic and Self-Cleaning Antifouling Properties

**DOI:** 10.3390/nano14020150

**Published:** 2024-01-10

**Authors:** Yannan Li, Yu Xue, Jie Wang, Dan Zhang, Yan Zhao, Jun-Jie Liu

**Affiliations:** School of Physical Science and Technology, College of Energy Materials and Chemistry, Inner Mongolia University, Hohhot 010021, China; liyannan@imu.edu.cn (Y.L.); 32146093@mail.imu.edu.cn (Y.X.); 32246101@mail.imu.edu.cn (J.W.); 32146053@mail.imu.edu.cn (D.Z.)

**Keywords:** self-cleaning, super-hydrophilic, oleophobic, zinc oxide, antibacterial

## Abstract

Super-hydrophilic and oleophobic functional materials can prevent pollution or adsorption by repelling oil, and have good circulation. However, traditional strategies for preparing these functional materials either use expensive fabrication machines or contain possibly toxic organic polymers, which may prohibit the practical application. The research of multifunctional ZnO microstructures or nanoarrays thin films with super-hydrophilic, antifouling, and antibacterial properties has not been reported yet. Moreover, the exploration of underwater oleophobic and self-cleaning antifouling properties in ZnO micro/nanostructures is still in its infancy. Here, we prepared ZnO microstructured films on fluorine-doped tin oxide substrates (F-ZMF) for the development of advanced self-cleaning type super-hydrophilic and oleophobic materials. With the increase of the accelerators, the average size of the F-ZMF microstructures decreased. The F-ZMF shows excellent self-cleaning performance and hydrophilic (water contact angle ≤ 10°) and oleophobic characteristics in the underwater antifouling experiment. Under a dark condition, F-ZMF-4 showed good antibacterial effects against *Staphylococcus aureus* (*S. aureus*) and *Escherichia coli* (*E. coli*) with inhibition rates of 99.1% and 99.9%, respectively. This study broadens the application scope of ZnO-based material and provides a novel prospect for the development of self-cleaning super-hydrophilic and oleophobic materials.

## 1. Introduction

After a long time of survival competition, the functions and structures of natural organisms have become perfect [1], for example, lotus leaves and dragonfly wings exhibit super hydrophobic structures, sharks display good anti-fouling properties, and purple leaf reed grass present super hydrophilicity. Inspired by the special wettability of natural organisms, researchers have designed several materials with unique surface structures, such as superlipophilic materials and superhydrophilic materials [2,3,4]. Superhydrophobic materials can be used for waterproofing, self-cleaning, anti-adhesion, reducing resistance, etc. However, some defects greatly hinder the realization of its function in practical applications, for instance, the surface of some superhydrophobic materials is easily absorbed by oil and has poor liquidity [5]. In contrast, the self-cleaning type super-hydrophilic and oleophobic functional materials can prevent pollution or adsorption by repelling oil, and have good circulation [6]. Nevertheless, the surface of general oleophobic materials is hydrophobic due to the larger surface energy of water than oil [7], so it is difficult to prepare self-cleaning type super hydrophilic and oleophobic functional materials. Moreover, pollution from bacteria in the water will affect or weaken the function of materials [8,9,10,11,12], thereby reducing their circularity. Therefore, it is urgent to develop self-cleaning type super-hydrophilic and oleophobic materials with advanced antibacterial, and antifouling properties through a high-efficiency and low-cost strategy.

In the past decades, researchers have found that super hydrophilic and oleophobic materials can be obtained by changing the surface structure and regulating its chemical composition [13,14,15,16,17]. Thus, the smart design and careful preparation of self-cleaning type super-hydrophilic and oleophobic materials play a vital role. Although a variety of materials have been reported [18,19,20,21,22], the scope of self-cleaning type super-hydrophilic and oleophobic materials should be branching out. More importantly, it is still significant to explore novel and excellent self-cleaning type super-hydrophilic and oleophobic materials.

Among various nanomaterials, zinc oxide (ZnO) with large specific surface area, fast electron transport rate, quantum finite domain effect, piezoelectric effect [23,24,25,26], and light diffraction has attracted great attention in the fields of nano-piezoelectric generators [27,28], dye-sensitized solar cells [29], quantum dot sensitized solar cells [30], and photoelectrochemical water decomposition [31]. ZnO has been used as a surface modifier to form fiber-based composite flexible membranes (such as carbon nanofibers, poly(arylene ether nitrile) fibrous membrane) for super-hydrophilic interface, oil-water separation, self-cleaning, antifouling, and so on [6,32,33]. Other researchers added ZnO as a filler combined with organic polymer materials to achieve superhydrophobic interfaces, antibacterial or photocatalytic self-cleaning properties [34]. However, the above strategies either use expensive fabrication machines or contain possibly toxic organic polymer materials, which may prohibit the practical application [35]. To our best knowledge, the research of multifunctional ZnO microstructures or nanoarrays thin films with super-hydrophilic, antifouling and antibacterial properties has not been reported yet [36]. Moreover, even different morphologies of ZnO (such as microrods, nanowires, nanotubes, nanosheets, nanoribbons, and their combined structures) prepared by different methods have been reported [37,38,39,40,41], the exploration of underwater oleophobic and self-cleaning antifouling properties in ZnO microstructures is still in its infancy possibly due to the difficulty in controlling morphology and the selective growth of substrate materials. In this paper, ZnO microstructured films (F-ZMF) with a flower-like structure were prepared by a simple one-step hydrothermal method using fluorine doped tin oxide (FTO) as a substrate and a mixture of zinc nitrate and hexamethylenetetramine solution as precursor solution. The microstructure of F-ZMF series samples is tuned by varying the concentration of HMTA. The water contact angle of F-ZMF series materials with super hydrophilicity is less than 10°, which shows excellent anti-fouling and self-cleaning properties underwater. In addition, F-ZMF-4 has a bacteriostatic effect of more than 99% against *Staphylococcus aureus* (*S. aureus*) and *Escherichia coli* (*E. coli*) under a dark condition, indicating an excellent antibacterial effect. This work provides a new prospect and an enlarged scope for the development of self-cleaning ZnO-based super-hydrophilic and oleophobic materials.

## 2. Materials and Methods

### 2.1. Materials

Zinc nitrate (Zn (NO_3_)_2_·6H_2_O, Tianjin Nankai share compounds Co., Ltd., Tianjin, China), hexamethyl tetramine (HMTA, Tianjin Nankai share compounds Co., Ltd., Tianjin, China), fluorine-doped tin oxide conductive glass (FTO, 1 × 1 cm^2^, Shenzhen South China Xiangcheng Technology Co. Ltd., Shenzhen, China), luria-bertani agar (LBA, Beijing Land Bridge Technology Co., Ltd., Beijing, China), Mueller-Hinton Broth (MHB, Beijing Land Bridge Technology Co., Ltd., Beijing, China), and phosphate-buffered saline (PBS, Beijing Kulaibo Technology Co., Ltd., Beijing, China) were of analytical grade and the experimental water was deionized water. Diesel and silicone oil are purchased from the local market in China.

### 2.2. Sample Preparation

During the preparation process, ZnO was prepared following the previous reported procedure with a some modification [33]. Firstly, an appropriate amount of Zn(NO_3_)_2_ and HMTA was added to 15 mL of deionized water, respectively, to prepare solution A (Zn(NO_3_)_2_ solution, 0.025 mol/L) and solution B (HMTA solution, 0.025 mol/L). Then, solutions A and B were fully mixed in proportion and added to a 50 mL hydrothermal reactor with FTO glass with conductive side up. After sealing, the reaction was carried out at 200 °C for 6 h. After the reaction, the sample is washed with deionized water and dried. Under the same experimental conditions, the molar concentration ratio of Zn (NO_3_)_2_ and HMTA was changed (1:0.5, 1:1, 1:2, 1:3, and 1:4), and the obtained samples were successively named F-ZMF-0.5, F-ZMF-1, F-ZMF-2, F-ZMF-3, and F-ZMF-4, respectively.

### 2.3. Characterization

The morphology and distribution of the materials were observed using a field emission scanning electron microscope (Hitachi, SU8010, Scanning Electron Microscope, SEM, Kyoto, Japan). X-ray diffraction analysis of the samples was performed using an X-ray diffractometer (RIKEN, Mini Flex 600, Diffraction of X-rays, XRD, Osaka, Japan) to determine the crystalline structure and orientation distribution of the samples. Point surface analysis was performed using an X-ray energy spectrometer (Bruker X Flash 6160, Energy Dispersive Spectroscopy, EDS, Karlsruhe, Germany) for qualitative analysis of all elements. The contact angle measuring instrument (SDC-350, Dongguan Shengding Precision Instrument Co., Ltd., Dongguan, China) was used to drop 2 μL of deionized water to measure the water contact angle on the surface of the material and take digital photos. Bacterial concentration was tested using a bacterial cell concentration meter (OD600, IMPLEN, Wetzlar, Germany). In this study, samples were tested at room temperature with an average power of around 100 mW by an in situ Raman spectrometer (HORIBA, LabRAM Odyssey, Kyoto, Japan) using a laser with a wavelength of 532 nm.

### 2.4. Surface Antibacterial Experiment

The antibacterial activity of the samples against *Escherichia coli* (*E. coli*) and *Staphylococcus aureus* (*S. aureus*) was detected by plate counting. The antibacterial experiments were carried out on an ultra-clean table, and the required supplies were autoclaved at 121 °C for 15 min before the experiments. Various samples of experimental synthesis were laid flat into 12-well plates, and the bacterial concentration OD was determined as *E. coli* 0.13 and *S. aureus* 0.06, which were diluted 10 times and 20 times, respectively. The 20 μL of diluted bacterial solution was evenly distributed on the surface of the samples and incubated at 37 °C for 2 h in a constant temperature incubator. Add the appropriate amount of PBS to wash the bacteria on the surface of the samples as the original bacterial solution, and after diluting again with PBS solution one by one, take 100 μL of this bacterial solution into a Petri dish and pour it into LBA medium, prepare agar plates of the corresponding samples for counting, and incubate them overnight at 37 °C in a bacterial incubator. The next day, count the number of colonies of each sample of the two bacteria and take representative photos. The antibacterial rate of the samples was calculated according to Equation (1)

(1) $$R=\frac{N_{0}-N_{t}}{N_{0}}*100\%$$

where *N*_0_, *N_t_*, are the number of surviving colonies on the surface of the control and experimental group samples, respectively.

### 2.5. Antifouling Test

This experiment tests the contact angle of water droplets or oil droplets on the surface of the sample and is used to analyze the wettability or anti-fouling of the sample surface. First, oil is selected as a pollutant, and the oil contact angle under the water is tested. The sample is then placed at the bottom of a beaker containing deionized water, a pipette is used to draw the 2 mL of oil and squeeze it onto the sample surface. Finally, the interaction between the sample and the oil droplets is observed and representative photos are taken. The corresponding Videos S1–S3 of the surface stability test, underwater antifouling test, and oil stain adhesion test of F-ZMF samples can be seen in the Supporting Information Part.

## 3. Results and Discussion

### SEM, XRD, and Surface Wettability Analysis of F-ZMF

**Figure 1:**
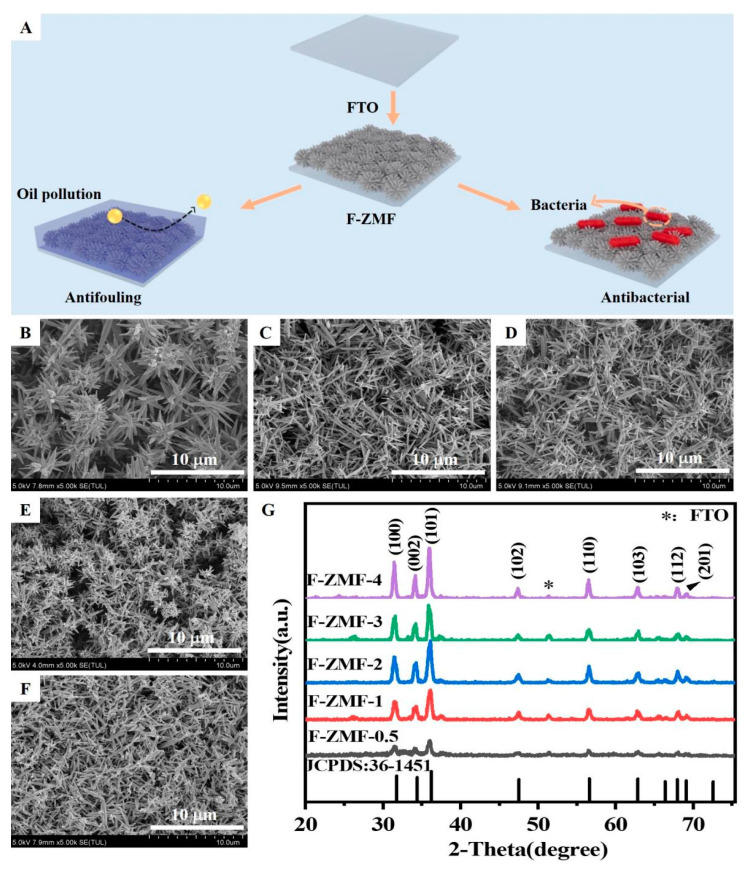
(**A**) Schematic diagram of antifouling and antimicrobial of F-ZMF. SEM images of (**B**) F-ZMF-0.5, (**C**) F-ZMF-1, (**D**) F-ZMF-2, (**E**) F-ZMF-3, (**F**) F-ZMF-4, (**G**) XRD patterns of F-ZMF samples.

Here, ZnO microstructured films (F-ZMF) were prepared on FTO substrate by a simple hydrothermal method, and their hydrophilic, oleophobic, and antibacterial properties were evaluated (Figure 1A). Compared with the native glass substrate, the surface of FTO has a certain roughness, which will affect the growth and orientation of ZnO and enhance the bonding degree between ZnO and the substrate surface [42]. When the concentration of HMTA is low, the reaction solution is weakly alkaline, and ZnO is easy to grow radially, thus forming a large, sparsely arranged flower-like structure. With the increase of HMTA concentration, a strong alkaline reaction solution was obtained, which inhibited the radial growth of ZnO, and more dense small microstructures were formed at multiple sites (Figure 1B–E). This result is also consistent with the growth mechanism of different ZnO crystal faces whose growth rates are affected by pH in literature [43].

The XRD patterns of F-ZMF samples are shown in Figure 1G. Three diffraction peaks with high intensity appeared at 30°~40°. The peak at 36.2° shows the highest intensity, indicating that the crystals preferentially grow along the direction of the (101) crystal plane. The obtained samples are mainly composed of ZnO microstructures because of the similar diffraction peaks of hexagonal ZnO (JCPDS:36-1451). It should be noted that the intensity of diffraction peaks is higher with the increase of HMTA concentration, indicating the significantly enhanced crystallization degree of the sample [44]. In addition, the EDS and atomic percentages of F-ZMF samples also confirm the successful synthesis of ZnO microrods (Figure S1). To further verify the difference among the F-ZMF samples, the normalized Raman spectra of the F-ZMF samples are shown in Figure S2A. All samples exhibit an intense peak at 438 cm^−1^ and two other peaks within the bands ranging from 200 to 800 cm^−1^. Similar to the XRD spectra, all peaks of the Raman spectra can be matched to the phonon modes of hexagonal wurtzite ZnO [45]. The peaks located at 331, 380, and 438 cm^−1^ correspond to the E2(high)−E2(low), A1(TO), and E2(high) phonon modes, respectively. Moreover, E2(high) modes of the samples shifted towards higher wave number (accounted for by oxygen vibrations) of approximately 1–2 cm^−1^ with the increase of HMTA concentration (Figure S2B), indicating the influence of amine molecules on the lattice vibrations. Our result is also consistent with the conclusions from other scientists. For example, Ngac et al. also reported three peaks of ZnO/Ag nanoflowers at 330, 376, and 437 cm^−1^, which are related to the vibrations modes of E2(high)−E2(low), A1(TO), and E2(high) [46]. The most prominent peak at 438 cm^−1^ is related to the vibration of zinc lattices [46,47,48].

**Figure 2:**
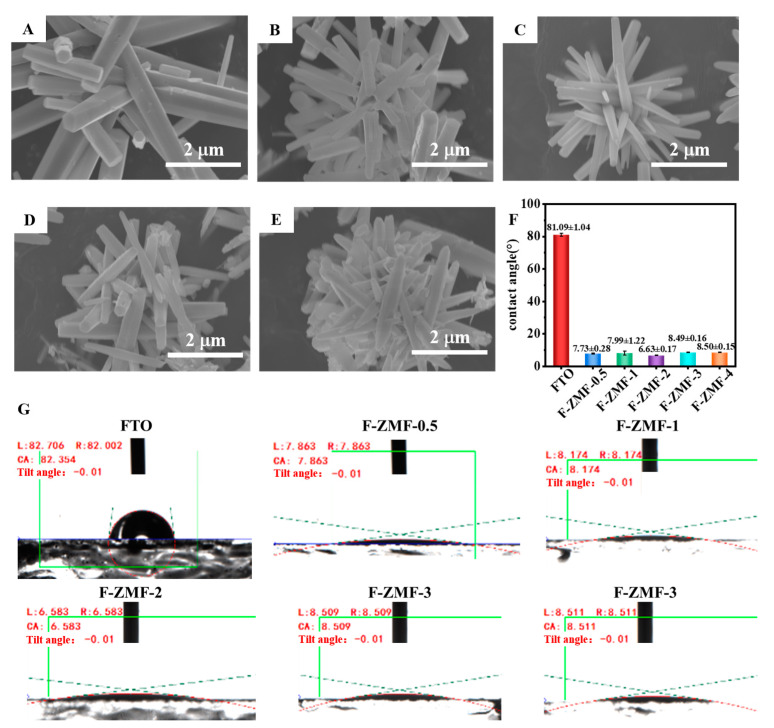
SEM images of (**A**) F-ZMF-0.5, (**B**) F-ZMF-1, (**C**) F-ZMF-2, (**D**) F-ZMF-3, (**E**) F-ZMF-4. Water contact angle (**F**) images and (**G**) histogram.

To further observe the microstructure of the samples, the morphologies of F-ZMF were observed by SEM images (Figure 2A–E). Scattered radiating rod-shaped ZnO structures can be observed, forming a multi-branched structure and a surface rich in microstructures. The formation of ZnO microrods is mainly due to the formation of crystal nuclei by Zn ions under the action of an alkaline reaction solution and surfactant. The microrods were grown along a specific crystal face direction under suitable reaction conditions. As a surfactant, HMTA can regulate the process of crystal seed formation and growth [45,49]. Increasing the concentration of HMTA will affect the chemical properties and surface tension of the solution, thus altering the growth and assembly of ZnO microrods. It has been confirmed that increasing the microstructure and specific surface area of materials can help improve the surface wettability and antibacterial properties of materials [50,51]. The diameters and lengths of zinc oxide were counted using an Image J 1.54g software based on the existing SEM images of F-ZMF. Because the structure of ZnO is thin at both ends and wide in the middle, the position with the widest ZnO structure was selected for measurement. The statistical histograms of the lengths and diameters of F-ZMF samples are shown in Figure S3. The diameters and lengths of F-ZMF with similar trends were inhibited with the increased concentration of HMTA in the reaction solution.

In Figure 2F,G, compared with the surface water contact angle of FTO (81.09°), the water contact angle of F-ZMF samples was less than 10°, showing superhydrophilicity. In Table S1, the area of water droplets diffused on the surface of the F-ZMF samples is 3.5–5 times that of the FTO surface. This implies that the superhydrophilicity of F-ZMF samples mainly comes from the diffusion of water droplets on the sample surface, rather than the penetration of microstructures. Due to the growth of ZMF structures on the surface of FTO, the water contact area and the wettability of samples were enhanced. In detail, because of the irregular accumulation of microrods, water droplets can spread on their surface and even penetrate the microstructure, forming a hydrophilic surface [52]. The crystal structure of ZnO contains both oxygen and zinc atoms, where the oxygen atoms form the oxide surface. The hydroxyl group (−OH group) on the surface of ZnO can form hydrogen bonds with water molecules [32], thus enhancing the interaction ability between ZnO and water. The unique interaction is beneficial to the formation of a small contact angle [53]. In that case, water molecules can be better wetted on the surface, thus achieving excellent hydrophilicity [32]. The F-ZMF samples exhibit good oil moisture properties and mechanical stability in air, as shown in Figures S4 and S5, and Video S1.

**Figure 3:**
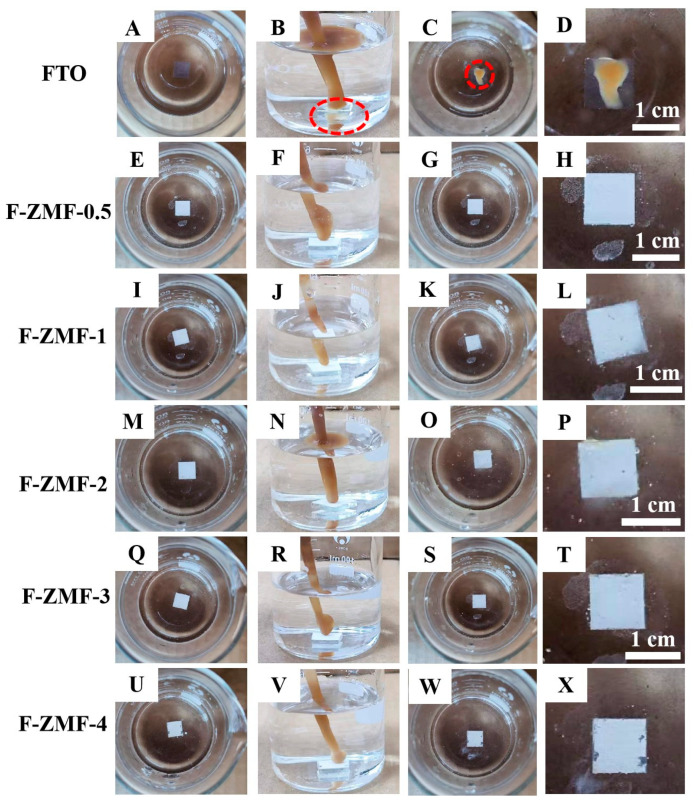
Optical pictures of F-ZMF during the self-cleaning experiments. (**A**,**E**,**I**,**M**,**Q**,**U**) the surface of the samples before contact with the oil droplets, (**B**,**F**,**J**,**N**,**R**,**V**) oil droplets in contact with different surfaces, (**C**,**G**,**K**,**O**,**S**,**W**) the surface of the sample after contact with oil droplets, (**D**,**H**,**L**,**P**,**T**,**X**) images of samples’ surfaces after contact with oil droplets.

It is well known that the hydrophilic properties caused by most physical structures are accompanied by the lipophobic properties of the structure surface, and F-ZMF samples are no exception [54,55]. In the underwater self-cleaning experiment, FTO was used as the control group and the F-ZMF sample as the experimental group. In Figure 3A–D, the oil adheres directly to the surface of FTO when it starts to contact with FTO. In comparison, no oil adhered when the oil droplets contact with the surfaces of F-ZMF (Figure 3E–X). The F-ZMF materials exhibit excellent oleophobic properties while being hydrophilic, and have excellent self-cleaning properties, which can be attributed to the difference of surface microstructure in F-ZMF materials. The video of the underwater antifouling test for the F-ZMF samples is also supplied and further confirms the stability of the samples (Video S2).

**Figure 4:**
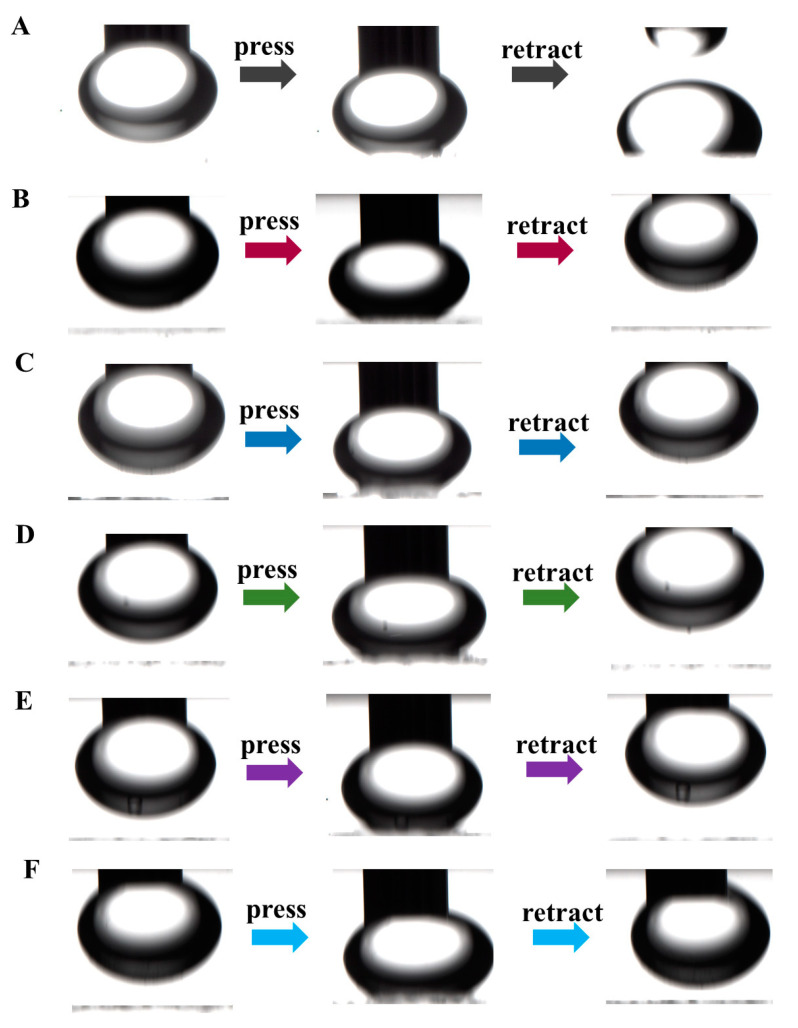
Underwater oil contact angle process of (**A**) FTO, (**B**) F-ZMF-0.5, (**C**) F-ZMF-1, (**D**) F-ZMF-2, (**E**) F-ZMF-3, (**F**) F-ZMF-4.

To verify the sample’s oleophobic properties, the underwater oil contact angle test was carried out (Figure 4 and Video S3). It can be observed from Figure 4A that when the silicone oil is in contact with the FTO surface underwater, it will adhere to the surface, at which time the oil contact angle is about 100.3°. When the silicone oil touches the surface of the F-ZMF series material, the oil droplets will float to the water surface due to their buoyancy (Figure 4B–F). As shown in Figure S6, the stability of F-ZMF was confirmed by optical images before and after the underwater oil thinning experiment. This phenomenon is consistent with the results of underwater self-cleaning. This indicates that F-ZMF behaves as a superoleophobic interface in aqueous media. The excellent oil rejection performance can be mainly attributed to the special surface structure of F-ZMF materials.

**Figure 5:**
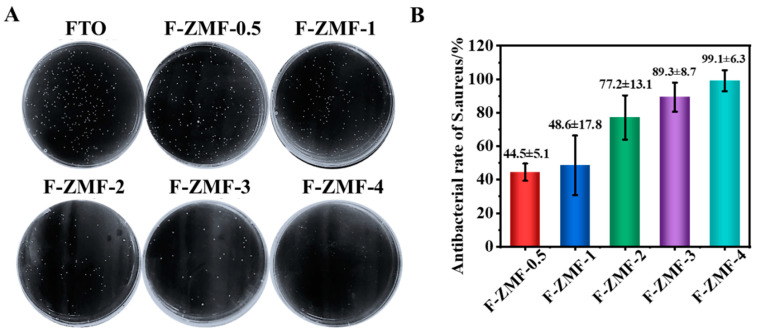
Antibacterial testing of different co-cultivation times of F-ZMF against *S. aureus*, (**A**) Plate counting pictures, (**B**) Surface antibacterial rate.

Under dark conditions, *S. aureus* and *E. coli* were selected to test the antimicrobial properties of F-ZMF materials, in which the pore plate was the control group and other F-ZMF materials were the experimental group. It can be seen in Figure 5A that with the increase of the HMTA addition ratio, the number of *S. aureus* colonies on the agar plate gradually decreased, indicating that the antibacterial performance was improving. Compared with the control group, the antibacterial activity of F-ZMF-0.5 reached 44.5%. With the increase of HMTA concentration, the antibacterial rate of F-ZMF-4 was significantly increased, reaching more than 99% (Figure 5B). This is because ZnO with more small-size microrods structure improves the contact area with bacteria, thus improving the antibacterial effect.

**Figure 6:**
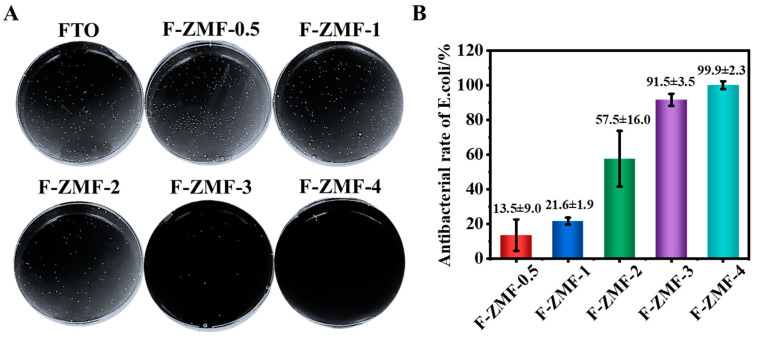
Antibacterial testing of different co-cultivation times of F-ZMF against *E. coli*, (**A**) Plate counting pictures, (**B**) Surface antibacterial rate.

**Figure 7:**
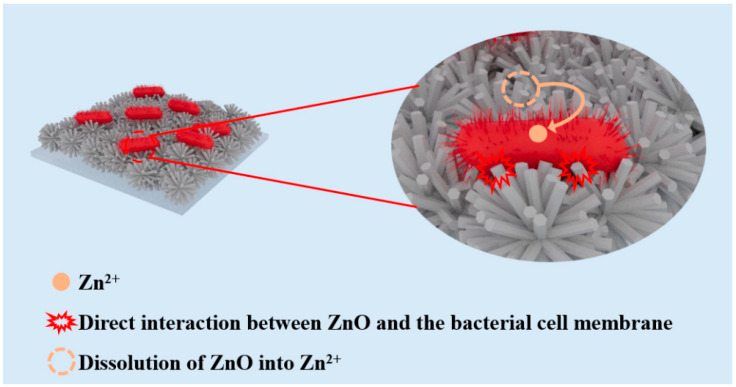
Schematic diagram of the antimicrobial mechanism of F-ZMF against bacteria.

Similar antibacterial activity has been observed in experiments with *E. coli*, as shown in Figure 6. Compared with the control group, the bacteriostatic rate of F-ZMF-4 could reach more than 99% due to the increased concentration of HMTA. The antibacterial effect of individual samples is slightly different, which may be due to the size effect of the material and bacteria. The F-ZMF samples with a smaller sharp tip of microrod and a larger contact area can cut through the cell membranes of bacteria, causing the rupture of membranes, the leakage of cell contents, and finally the death of bacteria cells [56]. At the same time, the released Zn^2+^ in the F-ZMF samples with bactericidal and antibacterial effects can destroy the bacterial cell membranes and the activity of intracellular enzymes, inhibiting bacterial growth and reproduction [56]. The combined actions make our samples excellent antibacterial activity (Figure 7).

## 4. Conclusions

In this paper, a series of multifunctional F-ZMF materials have been developed by a one-step hydrothermal method. SEM and XRD analysis confirmed that the material is mainly composed of evenly distributed hexagonal ZnO microrods with a typical hexagonal wurtzite structure. This material with a large surface contact area and special structural characteristics is beneficial to the improvement of the wettability. F-ZMF series samples exhibit excellent hydrophilic and underwater oil phobic properties, which is helpful in realizing surface self-cleaning performance. Profiting from the outstanding surface antibacterial performance (the antibacterial rate of some samples to *E. coli* and *S. aureus* exceeds 99% under dark conditions), our multifunctional F-ZMF materials can largely resist the pollution of bacteria, thus, achieving efficient and multi-faceted anti-fouling self-cleaning performance. This unique ZnO-based material may further broaden the applications for future self-cleaning, biome, and biomedicine areas.

## Data Availability

Data are contained within the article and supplementary materials.

## Supplementary Materials

The following supporting information can be downloaded at: https://www.mdpi.com/article/10.3390/nano14020150/s1, Figure S1. Energy spectra and the atomic percentages of different elements in (A) F-ZMF-0.5, (B) F-ZMF-1, (C) F-ZMF-2, (D) F-ZMF-3, (E) F-ZMF-4. Figure S2. (A) normal and (B) enlarged Raman spectra of the F-ZMF samples. Figure S3. Statistical histograms of (A) lengths and (B) diameters of F-ZMF. Figure S4. Silicone oil contact angle of F-ZMF. Figure S5. Diesel oil contact angle of F-ZMF. Figure S6. Optical images of the samples before and after the underwater oleophobic experiment. Table S1. Water droplet diffusion area of different samples. Video S1. Surface stability test of F-ZMF. Video S2. Underwater antifouling test of F-ZMF. Video S3. Oil stain adhesion test of F-ZMF (taken with the contact angle tester underwater condition).
