# Supplementary material for: Antibacterial Hydrophilic ZnO Microstructure Film with Underwater Oleophobic and Self-Cleaning Antifouling Properties
Source: Nanomaterials (Basel). 2024 Jan 10;14(2):150. doi: 10.3390/nano14020150 (PMC10820557; doi:10.3390/nano14020150)
Supplement: Supplementary file 1 [file nanomaterials-14-00150-s001.zip › nanomaterials-2753563-supplementary.pdf]

Supporting information

Antibacterial Hydrophilic ZnO Microstructure Film with Underwater Oleophobic and Self-Cleaning Antifouling Properties

Yannan Li, Yu Xue, Jie Wang, Dan Zhang, Hongfei Dou, Musen Du, Yan Zhao * and Jun-Jie Liu *

School of Physical Science and Technology, College of Energy Materials and Chemistry, Inner Mongolia University, Hohhot 010021, China; liyannan@imu.edu.cn (Y.L.); 32146093@mail.imu.edu.cn (Y.X.); 32246101@mail.imu.edu.cn (J.W.); 32146053@mail.imu.edu.cn (D.Z.); 32346098@mail.imu.edu.cn (H.D.); 32346108@mail.imu.edu.cn (M.D.)

* Correspondence: yanzhao@imu.edu.cn (Y.Z.); pyljj@imu.edu.cn (J.-J.L.)

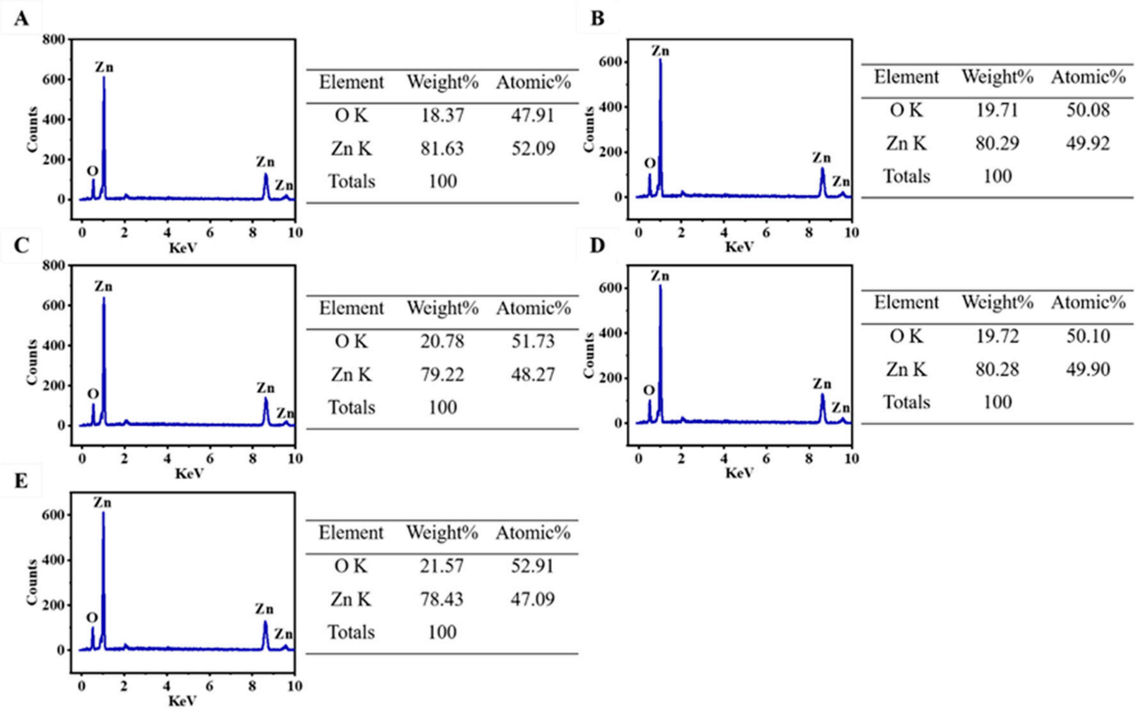

(A)

Element	Weight%	Atomic%
O K	18.37	47.91
Zn K	81.63	52.09
Totals	100	

(B)

Element	Weight%	Atomic%
O K	19.71	50.08
Zn K	80.29	49.92
Totals	100	

(C)

Element	Weight%	Atomic%
O K	20.78	51.73
Zn K	79.22	48.27
Totals	100	

(D)

Element	Weight%	Atomic%
O K	19.72	50.10
Zn K	80.28	49.90
Totals	100	

(E)

Element	Weight%	Atomic%
O K	21.57	52.91
Zn K	78.43	47.09
Totals	100	

Fig. S1 Energy spectra and the atomic percentages of different elements in (A) F-ZMF-0.5, (B) F-ZMF-1, (C) F-ZMF-2, (D) F-ZMF-3, (E) F-ZMF-4.

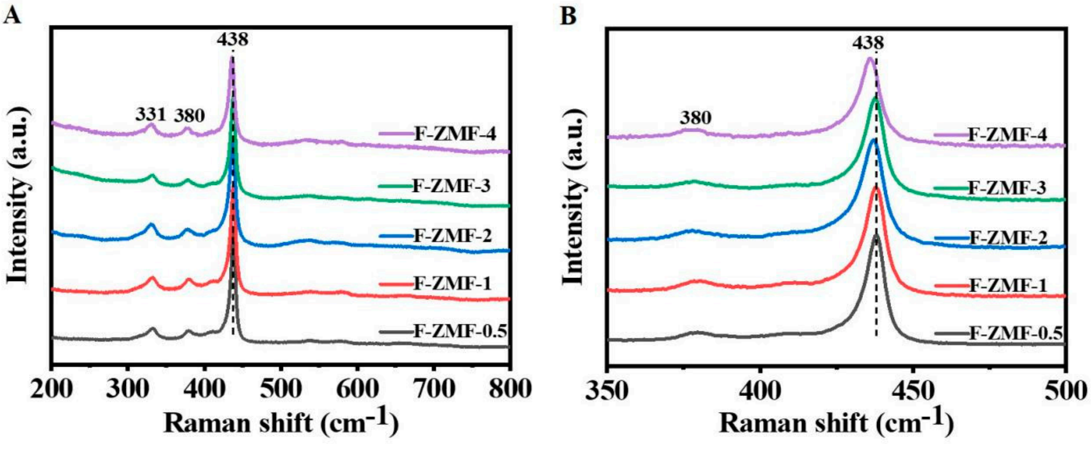

Fig. S2 (A) normal and (B) enlarged Raman spectra of the F-ZMF samples.

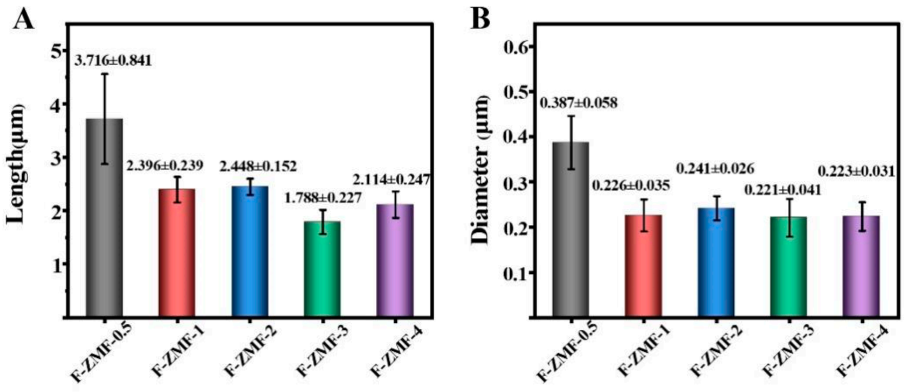

Fig. S3 Statistical histograms of (A) lengths and (B) diameters of F-ZMF.

Table S1 Water droplet diffusion area of different samples

	FTO	F-ZMF-0.5	F-ZMF-1	F-ZMF-2	F-ZMF-3	F-ZMF-4
Droplet diffusion diameter (mm)	1.87	4.16	3.46	4.05	3.83	3.78
Droplet diffusion area （mm^2）	2.74	13.61	9.41	12.86	11.51	11.21

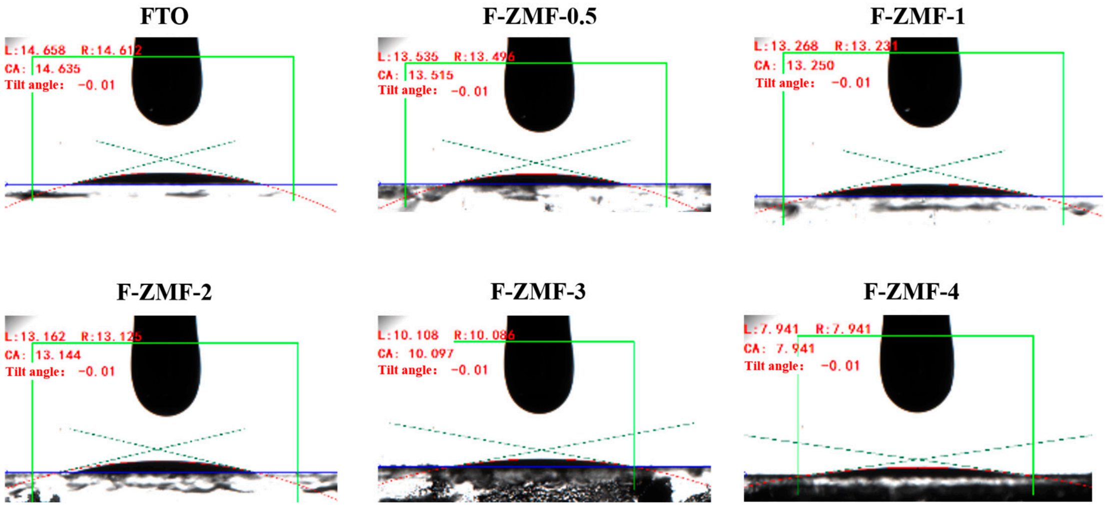

Fig. S4 Silicone oil contact angle of F-ZMF.

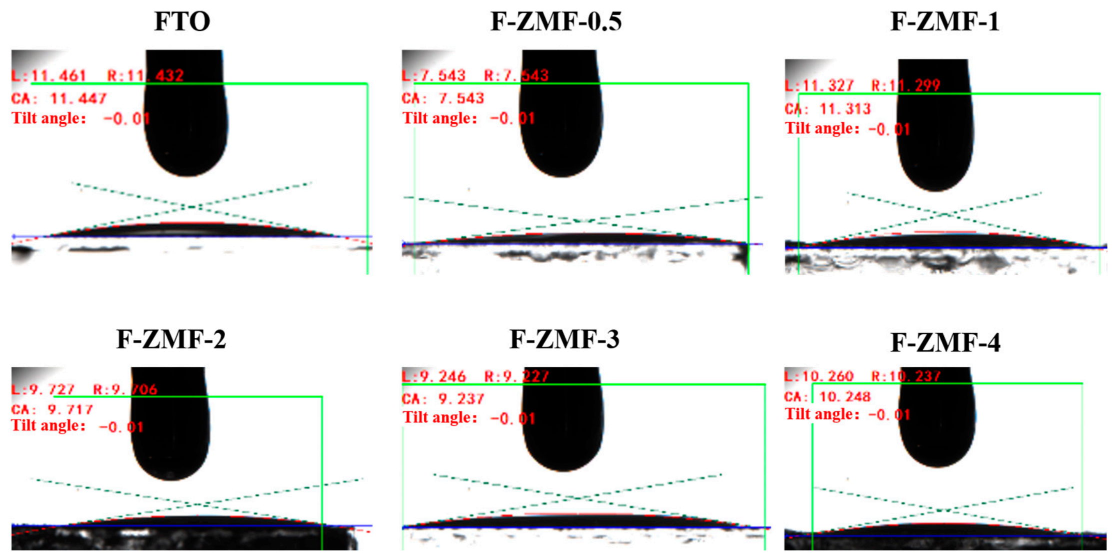

Fig. S5 Diesel oil contact angle of F-ZMF.

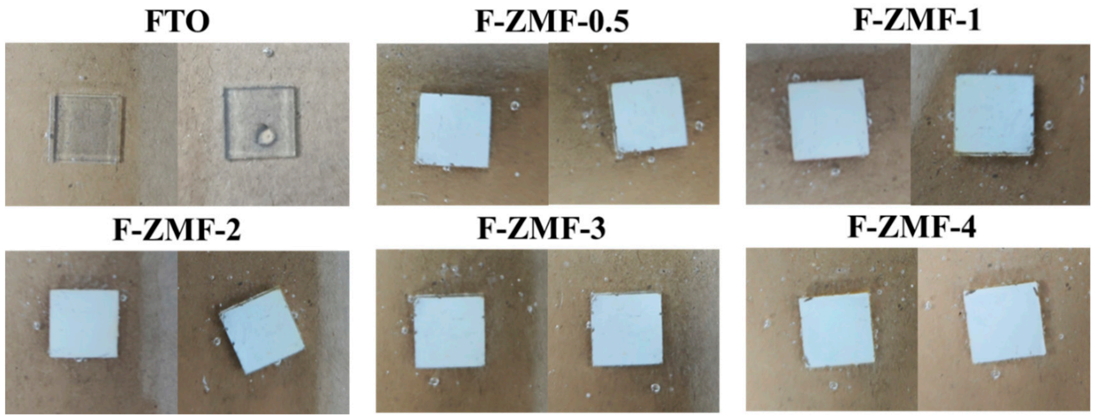

Fig. S6 Optical images of the samples before and after the underwater oleophobic experiment.

Video S1 Surface stability test of F-ZMF.

Video S2 Underwater antifouling test of F-ZMF.

Video S3 Oil stain adhesion test of F-ZMF (taken with the contact angle tester underwater condition).
